# Microbiota of the prostate tumor environment investigated by whole-transcriptome profiling

**DOI:** 10.1186/s13073-022-01011-3

**Published:** 2022-01-25

**Authors:** Paul Vinu Salachan, Martin Rasmussen, Jacob Fredsøe, Benedicte Ulhøi, Michael Borre, Karina Dalsgaard Sørensen

**Affiliations:** 1 Department of Molecular Medicine, Aarhus University Hospital, 8200 Aarhus N, Denmark; 2 Department of Clinical Medicine, Aarhus University, 8200 Aarhus N, Denmark; 3 Department of Pathology, Aarhus University Hospital, 8200 Aarhus N, Denmark; 4 Department of Urology, Aarhus University Hospital, 8200 Aarhus N, Denmark

**Keywords:** Prostate cancer, Microbiome, Metatranscriptome, *Vibrio parahaemolyticus*, *Microbacterium sp*., *Shewanella*, Dendritic cells

## Abstract

**Background:**

With over 350,000 estimated deaths worldwide in 2018, prostate cancer (PCa) continues to be a major health concern and a significant cause of cancer-associated mortality among men. While cancer in general is considered a disease of the human genome, there is a growing body of evidence suggesting that changes to the healthy microbiota could play a vital role in cancer development, progression, and/or treatment outcome.

**Methods:**

Using a metatranscriptomic approach, we annotated the microbial reads obtained from total RNA sequencing of 106 prostate tissue samples from 94 PCa patients (discovery cohort). We investigated microbial dysbiosis associated with PCa by systematically comparing the microbiomes between benign and malignant tissue samples, between less vs. more-aggressive PCa, and between patients who had biochemical recurrence as opposed to those who did not. We further performed differential gene expression and cell type enrichment analysis to explore the host transcriptomic and cellular responses to selected microbial genera. A public dataset (GSE115414) of total RNA sequencing reads from 24 prostate tissue samples (8 benign and 16 malignant) served as the validation cohort.

**Results:**

We observed decreased species diversity and significant under-representation of *Staphylococcus saprophyticus* and *Vibrio parahaemolyticus*, as well as significant over-abundance of *Shewanella* in malignant as compared to benign prostate tissue samples in both the discovery (*p* < 0.01) and validation (*p* < 0.05) cohorts. In addition, we identified *Microbacterium* species (*p* < 0.01) to be significantly over-abundant in pathologically advanced T3 tumors compared to T2 in the discovery cohort. Malignant samples having high vs. low *Shewanella* counts were associated with downregulated Toll-like receptor signaling pathways and decreased enrichment of dendritic cells. Malignant samples having low vs. high *V. parahaemolyticus* counts were enriched for olfactory transduction and drug metabolism pathways. Finally, malignant samples were enriched for M1 and M2 macrophages as compared to benign tissue samples.

**Conclusions:**

The results from this exploratory study support the existence of an important biological link between the prostate microbiota and PCa development/progression. Our results highlight *Shewanella*, *V. parahaemolyticus*, and *Microbacterium sp.* as interesting candidates for further investigation of their association with PCa.

**Supplementary Information:**

The online version contains supplementary material available at 10.1186/s13073-022-01011-3.

## Background

Prostate cancer (PCa) remains the most prevalent cancer among men in Denmark and the second most incident cancer among men worldwide [1]. While mortality rates associated with PCa have shown a declining trend in some European countries [2], PCa still contributes to a significant fraction of global cancer mortality among men [1] with a continued rise globally due to population growth and increasing population age [1, 2].

While major risk factors for PCa include ethnicity, age, genetic predisposition, and a family history of PCa [3, 4], the role of inflammation in relation to PCa development and progression has also garnered scientific attention (reviewed in [5, 6]). Given the intricate relationship between inflammation and the host microbiota [7], the human microbial ecosystem is increasingly being implicated in the occurrence of various cancers with causal relationships reported in a few instances, e.g., *Helicobacter pylori* infection and gastric carcinoma [8].

Cancer tissue may also harbor unique microbial signatures that could be of diagnostic, prognostic, and/or therapeutic potential. For example, a recent large-scale study [9] found microbial signatures in tumors in several cancer types (e.g., stomach and lung adenocarcinomas) that were unique to each. Few earlier and smaller-scale studies specifically investigating microbial signatures in benign and malignant prostate tissue have also found some evidence to support a PCa-specific microbiota [10, 11]. However, other small-scale studies have failed to clearly discriminate benign vs. malignant prostate tissues based on either metagenomic or metatranscriptomic data [12], suggesting considerable variation in the presence of microbial signatures between patient cohorts. For example, it has been reported that prostate tumor tissue from African patients might harbor different microbial loads compared to Australian patients [13]. Nevertheless, all these studies support the existence of a non-sterile prostate microenvironment.

Currently, although no single pathogenic species has been implicated in PCa development, it is believed that microbial dysbiosis (i.e., changes to the healthy microbiota) could play a significant role in disease occurrence, progression, and/or treatment outcome [14], even though this area is still understudied. Hence, in order to study any potential dysbiosis associated with PCa development and progression, we analyzed the microbiome of benign (adjacent normal) and malignant (tumor) prostate tissue samples from a total of 94 Danish men with PCa. Using total RNA sequencing (RNAseq), we obtained and annotated microbial reads from all samples and systematically compared the microbiomes between benign and malignant tumor tissue, less vs. more-aggressive PCa, and between patients who had biochemical recurrence (BCR) as opposed to those who did not (BCR-free). We further characterized the host transcriptional regulation in response to increased/decreased abundance of specific organisms within the malignant tissue samples and subsequently performed cell type enrichment to explore differences in cellular composition within malignant tissue samples having high vs. low count of specific organisms.

## Methods

### Patient cohorts

Our PCa discovery cohort consisted of 114 prostate tissue samples from 102 patients who underwent curatively intended radical prostatectomy (RP) for histologically verified, clinically localized PCa, at the Department of Urology, Aarhus University Hospital (AUH), Denmark (2004–2017). Immediately following surgery, fresh tissue biopsies were obtained and stored at −80°C in TissueTek until further processing. Prior to RNA extraction, all tissue specimens were marked as either benign or malignant (PCa) based on histopathological examination of the H&E stained tissue sections by an experienced pathologist at AUH. Eight malignant tissue samples were excluded due to very high unmapped read counts, contributed primarily by known contaminant taxa [15]. Thus, the final cohort consisted of 106 tissue samples (23 benign and 83 malignant) from 94 patients (Additional file 1: Fig. S1). Clinical follow-up for this cohort was updated in November 2019.

For validation, we used a publically available dataset of total RNA sequencing data from a small cohort of PCa patients recruited in France [16, 17]. Collection of benign and tumor biopsy specimens was done retrospectively from the patients who gave informed consent. The cohort consisted of 24 prostate tissue samples (8 benign and 16 malignant) from men who underwent radical prostatectomy (Additional file 1: Fig. S1). Formalin-fixed paraffin-embedded tissue samples were used for total RNA extraction and sequencing. Clinicopathological characteristics for the discovery cohort and the validation cohort are given in Table 1.

**Table 1:** Clinicopathological and sequencing characteristics

**Clinicopathological characteristics**		
**Characteristic**	**Discovery cohort**	**Validation cohort**
Patients, *N*	94	24
Tissue samples, *N*	106	24
Age at RP, median (range)	65.7 (45.7–76.6)	N/A
**Tumor status**		
Benign, *N* (%)	23 (21.7%)	8 (33.3%)
Malignant, *N* (%)	83 (78.3%)	16 (66.6%)
**Pathological Gleason score**		
≤ 7, *N* (%)	63 (75.9%)	9 (56.2)
> 7, *N* (%)	20 (24.1%)	7 (43.7)
**Pre-operative PSA (ng/ml)**		
≤ 10, *N* (%)	39 (47%)	N/A
>10, *N* (%)	39 (47%)	N/A
Unknown, *N* (%)	5 (6%)	N/A
**Pathological T-stage**		
pT2, *N* (%)	51 (61.4%)	4 (25%)
pT3, *N* (%)	31 (37.4%)	10 (62.5%)
Unknown/pT4, *N* (%)	1 (1.2%)	2 (12.5%)
**Biochemical recurrence status**		
BCR, *N* (%)	28 (33.7%)	6 (37.5%)
BCR-free, *N* (%)	53 (63.9%)	10 (62.5%)
Unknown, *N* (%)	2 (2.4%)	--
**Median follow-up, months (range)**	52.4 (17.6–178.9)	N/A
**Sequencing characteristics**		
**Characteristic**	**Discovery Cohort**	**Validation cohort**
Total reads in bam per sample, mean (SD)	76,688,094 (11,296,752)	304,920,301 (49,502,192)
Reads per sample mapped to hg38, mean (SD)	60,131,687 (7,869,239)	170,390,820 (16,853,739)
Unmapped read pairs per sample, mean (SD)	970,526 (280,812)	2,734,147 (421,428)

*RP* radical prostatectomy, *PSA* prostate-specific antigen, *SD* standard deviation, *Hg* human genome

### Total RNA extraction and sequencing

For the discovery cohort, total RNA was extracted from fresh-frozen prostate tissue samples using the RNeasy Plus Mini Kit (Qiagen). The concentration and quality of RNA was assessed using a NanoQuant and an Agilent 2100 Bioanalyzer (RIN≥7), respectively. Sequencing libraries were generated using either the ScriptSeq RNA-seq Library Kit with the Ribo-Zero™ Magnetic Gold Kit from Illumina (37 malignant and 5 benign samples) or the KAPA RNA HyperPrep Kit with KAPA RiboErase Kit from Roche (46 malignant and 18 benign samples). All libraries were sequenced using Illumina NovaSeq or NextSeq 500. All reads were QC checked and aligned to the human reference genome hg38 using STAR [18]. On average, each sample bam file contained approximately 76 million 75bp reads (38 million read pairs) and approximately 60 million reads per sample aligned to hg38. We obtained approx. 1 million read pairs per sample that had both the mate pairs unmapped which we used for microbial read processing (Table 1).

For the validation cohort [16], we downloaded a public dataset of total RNA sequencing reads from the gene expression omnibus portal (accession number GSE115414 [17]). Alignment of the reads was performed as described above. On average, each sample bam file contained approximately 300 million reads and approximately 170 million reads per sample aligned to hg38. We obtained approx. 2.7 million read pairs per sample that had both the mate pairs unmapped which we used for microbial read processing (Table 1).

### Host read processing

Host transcript levels were quantified using kallisto [19] with hg38 as the reference transcriptome. Transcripts were aggregated to gene level using tximport [20]. Batch effects were either adjusted for in the design formula (for differential gene expression analyses) or removed using the RemoveBatchEffect function (for cell type enrichment analyses) within Limma [21].

### Microbial read processing

We followed a modified version of the SAMSA2 pipeline [22] for microbial read processing and differential microbial abundance analysis. Briefly, fastq files of reads that did not map to the human reference genome hg38 were generated from bam files using BEDTools [23] and thereafter paired-end reads were merged using PEAR [24]. DIAMOND [25] sequence aligner was used to align and annotate the unmapped reads against NCBI RefSeq bacterial non-redundant protein sequences database [26]. For annotation against viral sequences, additional viral protein sequences were downloaded from NCBI RefSeq database [26]. Custom python and R scripts provided with the SAMSA2 pipeline [22] were used for aggregating and merging the annotation files. The aggregated files with microbial read counts were subsequently used for downstream analysis.

### Comparison of overall microbial diversity

For both cohorts, we compared the microbial species diversity within malignant and benign prostate tissue samples by estimating the *alpha* diversity as implemented in the R phyloseq [27] and vegan [28] packages. Alpha diversity measures the total number of species (i.e., species richness) and their relative proportions in a population (i.e., evenness). Accordingly, differences in alpha diversities between malignant and benign prostate tissue samples would signify microbial dysbiosis and could be associated with disease occurrence. For visualizing alpha diversity, we used six different measures: Observed, Chao1, and ACE capture aspects of species richness, whereas Shannon, Simpson, and Inverse Simpson capture both species richness and evenness [29–31]. A Wilcoxon rank sum test was used to compare each diversity estimate between the two groups. *P* values were corrected for family wise error rate using the Bonferroni method [32] and significance was determined at a *p* value cut-off less than 0.05. We consider differences in alpha diversity between groups to be reliable only if the Shannon, Simpson, and Inverse Simpson diversity estimates showed a significant difference as these are more robust measures of alpha diversity [33].

In addition, alpha diversity was also estimated for the samples in the comparisons between low vs. high Gleason scores, low vs. high pre-operative Prostate-Specific Antigen (pre-op PSA) levels, pathological T-stage 2 vs. 3, and BCR vs. BCR-free groups for the discovery cohort (Additional file 1: Fig. S1). Due to the limited sample size, we did not include these analyses for the validation cohort.

### Differential microbial abundance analysis

Differential abundances between malignant (*n* = 83) and benign prostate tissue samples (*n* = 23) of microbial counts (as well as for other comparisons) in the discovery cohort were tested using the DESeq2 package [34] in R, and *p* values were adjusted for multiple testing using Benjamini-Hochberg [35] corrections. We used an adjusted *p* value less than 0.01 to make statistical inferences, as this would allow us to make five comparisons if the original significance level was at 0.05 (accounting for family-wise error rate from multiple comparisons made on the same dataset). Further, species having a *p* value less than 0.01 were considered to be significantly over-represented if they had a log_2_ fold change greater than 0.58 (corresponding to a fold change of 1.5) in the given comparison and under-represented if the log_2_ fold change was less than −0.58 in any given comparison. In order to avoid potential biases from species with low read counts, we filtered out the low abundance species having a mean normalized count of fewer than 10 across all samples. Since our samples were prepared using two different RNAseq library preparation kits, we accounted for batch effects in the differential abundance analysis by including the batch factor in the design formula.

In order to investigate microbial dysbiosis in less vs. more-aggressive PCa, we systematically compared malignant tissue samples from patients having low (≤ 7, *n* = 63) vs. high (>7, *n* = 20) Gleason scores, low (≤ 10 ng/ml, *n* = 39) vs. high (> 10 ng/ml, *n* = 39) pre-operative Prostate-Specific Antigen (pre-op PSA) levels, and a pathological T-stage 2 (pT2, *n* = 51) vs. 3 (pT3, *n* = 31). We further looked for differences in microbial abundance in patients who had BCR (*n* = 28) after RP compared to those who did not (BCR-free, *n* = 53). Postoperative BCR was defined as a PSA ≥ 0.2 ng/ml. Patients not suffering BCR were censored at their last normal PSA measurement. An overview of the different comparisons is given in Additional file 1: Fig. S1.

To validate the differentially abundant organisms identified between malignant and benign prostate tissue samples in the discovery cohort, we compared their differential abundance between malignant (*n* = 16) and benign prostate tissue samples (*n* = 8) in an independent set of samples (validation cohort). Due to the limited sample size (and composition) in this validation cohort, we could not test for differential abundance between less vs. more-aggressive PCa.

### Differential host transcriptional regulation

For the species identified as differentially over/less abundant in the malignant as compared to the benign tissue samples in both the cohorts, we performed host differential gene expression (DGE) analyses for elucidating the host transcriptional regulation in response to high (*n* = 42) or low (*n* = 41) species counts. Low species count was defined as a mean normalized count less than the median count. DGE analyses were performed similar to the methodology described above using the DESeq2 package [34] in R, however, with a significance cut-off of adjusted *p* value less than 0.05. Genes identified as differentially upregulated or downregulated were each used for Kyoto Encyclopedia of Genes and Genomes (KEGG) pathway analyses using DAVID bioinformatics resources [36]. A false discovery rate (FDR) less than 0.05 was used as a cut-off for determining significant pathways.

### Cell type enrichment analyses

For determining the cellular composition of the malignant tissue samples harbouring high (*n* = 42) or low (*n* = 41) counts of the species identified as either differentially over-abundant or differentially less abundant in the malignant samples in both the cohorts, we performed cell type enrichment analyses using xCell [37] in R for the tissue samples in the discovery cohort. Using this approach, we explored the stromal, epithelial, and immune cell types within the malignant tissue samples. Associations between the species abundance and the host cell types were tested using a Wilcoxon test with an FDR cut-off less than 0.05 used for determining significant associations.

Similarly, we also explored the host cellular composition between benign (*n* = 23) and malignant (*n* = 83) tissue samples using xCell, in order to find associations between specific immune/stromal cell types and PCa, which in turn may be linked with microbial dysbiosis in PCa.

### PCR validation of selected species

To validate the presence of microbial species within tissue samples, we selected *Bacteroides fragilis* as a candidate organism, due to its frequent presence in prostate tissue samples as identified from our microbiome analysis. Primers specific to *B. fragilis* were selected from the literature [38]. RNA extracted from 6 fresh-frozen RP tissue samples (3 malignant and 3 benign) was reverse transcribed using a mix of oligo dT and random hexamer primers. cDNA was amplified by PCR with a total reaction volume of 15 μl as follows: initial denaturation at 95°C for 5 min followed by 35 cycles of 94°C for 30s, 50°C for 1 min, and 72°C for 1 min. A final extension was performed at 72°C for 10 min. The PCR product was run on a 1% agarose gel, and the presence of a 495-bp amplification product was verified using a gel doc system. Next, 10μl PCR product was used for PCR clean-up and subsequent Sanger sequencing to verify the *B. fragilis* sequence. Forward and reverse reads from the sequencing were assembled into contigs using GeneStudio. Local alignment search against 16s ribosomal RNA sequences was performed using NCBI BLAST [39] optimized to retrieve highly similar sequences (megablast). Uncultured and environmental sample sequences were excluded from the search.

## Results

For metatranscriptomic analyses, we used a set of total RNA sequencing data from benign and malignant prostate tissue samples, annotated against known microbial reads in NCBI. The dataset included 106 tissue samples from 94 PCa patients (discovery cohort, Additional file 1: Fig. S1). The median patient age at RP was 65.7 years. Almost 76% of the tissue samples had a Gleason score ≤ 7 and 24% had a score greater than 7. An equal representation of samples had a pre-op PSA value ≤ 10 (47%) and > 10 ng/ml (47%), whereas approx. 61% and 37% of the samples had a pathological T-stage of pT2 and pT3, respectively. Almost 64% of the samples were from patients without BCR, while approx. 34% experienced BCR (median follow-up of 52.4 months). Using this discovery cohort, we compared differences between benign (*n* = 23) and malignant prostate tissue (*n* = 83) microbial profiles. We also systematically compared microbial species associations with more/less aggressive PCa using key clinicopathological factors known to be associated with PCa aggressiveness (pre-operative PSA level, Gleason score, T-stage) as well as post-operative BCR status (Table 1, Additional file 1: Fig. S1). Finally, for the species identified as differentially over/less abundant in the malignant as compared to the benign tissue samples, we performed host differential gene expression (DGE) analyses for elucidating the host transcriptional regulation in response to high (*n* = 42) vs. low (*n* = 41) species counts, followed by cell type enrichment analyses for exploring the differences in the cellular composition of the prostate tumor microenvironment (TME). Cell type enrichment analysis was also performed for comparing cellular differences between benign (*n* =23) and malignant (*n* =83) tissue samples.

### The microbial environment in primary prostate tumor tissue (radical prostatectomy specimens)

We first investigated the microbial species that were most abundant in primary prostate tissue samples from patients with PCa in the discovery cohort. We included both benign (*n* = 23) and malignant (*n* = 83) prostate tissue samples in order to get a first impression of the species that were predominant in prostate tissue samples from our cohort of Danish men who had undergone radical prostatectomy. Using the full sample set (*n* = 106), we found that the most abundant microbial reads belonged to *Enterobacter hormaechei*, accounting for 23.9% of the microbial reads in all the samples (Table 2). Other highly abundant microbial reads belonged to *Streptococcus pneumoniae* (6.9%), *Acinetobacter baumannii* (6.4%), *Mycobacterium sp.* (5.8 %), *Salmonella enterica* (5.4%), *Escherichia coli* (3.7%), *Campylobacter jejuni* (3.6%), *Clostridioides difficile* (3.6%), *Mycobacterium abscessus* (3.4%), and *Bacillus cereus* (1.1%) (Table 2)*.* However, seven genera (*Acinetobacter*, *Enterobacter*, *Streptococcus*, *Escherichia*, *Bacillus*, and *Mycobacterium*) of the 10 highly abundant species that we identified (Table 2) are known to be common contaminants in sequencing-based microbiome studies [15, 40, 41]. The remaining three of the highly abundant species that we detected (*S. enterica*, *C. jejuni*, and *C. difficile*) are not known to be contaminants, and hence likely reflect the “true” prostate microbial ecosystem. While we were able to detect viral species in the prostate tissue samples, we did not observe any viruses with high relative abundances across all samples, corroborating previous research [13] and suggesting that viruses are not predominant members of the prostate microbial ecosystem.

**Table 2:** Most abundant microbial species in the prostate tissue of patients who had PCa

Species	Total reads across all samples (***n*** = 106)	Relative proportions (% of all reads)	Mean number of reads per sample	Standard deviation
Enterobacter hormaechei*	674,201	23.9	6360.3	2749.8
Streptococcus pneumoniae*	195,368	6.9	1843.1	1185.9
Acinetobacter baumannii*	181,489	6.4	1712.1	2856.3
Mycobacterium sp.*	164,496	5.8	1551.8	1174.1
*Salmonella enterica*	153,667	5.4	1449.6	1078.6
Escherichia coli*	104,243	3.7	983.42	913.3
*Campylobacter jejuni*	102,543	3.6	967.3	515.7
*Clostridioides difficile*	102,525	3.6	1235.2	4082.6
Mycobacterium abscessus*	98,079	3.4	925.2	476.9
Bacillus cereus*	33,484	1.1	1455.8	883.4
Other	1,002,742	35.6	9459.8	5067.9

*Genera of these species are known to be common contaminants in sequencing based microbiome studies

### Microbial species diversity between benign and malignant PCa

Next, using six different measures of *alpha* diversity, we investigated overall differences in species richness (i.e., total number of species: Observed, Chao1, and ACE) and their evenness (i.e., relative abundances: Shannon, Simpson, and Inverse Simpson) between benign (*n* = 23) and malignant prostate tissue samples (*n* = 83) in the discovery cohort. Differences in alpha diversity between benign and malignant prostate tissue samples would indicate microbial dysbiosis that could be associated with PCa.

**Fig. 1:**
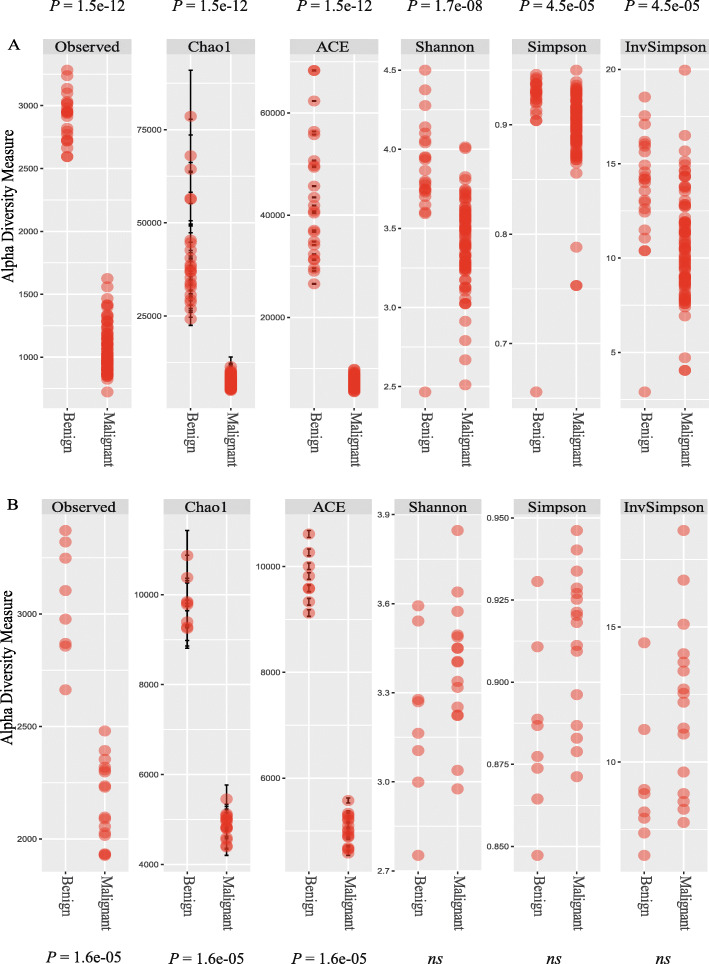
Differences in overall alpha diversity between benign and malignant tissue samples as measured using six different measures of alpha diversity for the discovery (**A**) and validation (**B**) cohorts. Observed, Chao1, and ACE capture aspects of species richness, whereas Shannon, Simpson, and Inverse Simpson capture both species richness and evenness. A Wilcoxon rank sum test was used to compare the diversity estimates between the two groups. Compared to the benign, malignant tissue samples showed an overall reduction in the species diversity in the discovery cohort which was significant at a *p* value cut-off of 0.05. While this trend was also observed in the validation cohort, the *p* values did not reach statistical significance for some of the diversity estimates. ns not significant

Visual inspection of the data suggested an overall reduction in species diversity in malignant tissue compared to benign tissue across all alpha diversity measures (Fig. 1A). A Wilcoxon rank sum test showed that these differences were also statistically significant for all the diversity measures (Additional file 1: Table S1), indicating decreased species richness and evenness in malignant tissue as compared to benign prostate tissue. A similar trend with an overall reduction in species diversity in the malignant (*n* = 16) compared to the benign tissue (*n* = 8) was also observed in the smaller validation cohort (Fig. 1B), although the differences were statistically significant for only Observed, ACE and Chao1 (Additional file 1: Table S1). Nevertheless, an overall reduction in the species diversity within malignant as compared to benign tissue samples indicates microbial dysbiosis associated with PCa development/progression.

### Differential abundance of species in benign vs. malignant prostate tissue samples

**Fig. 2:**
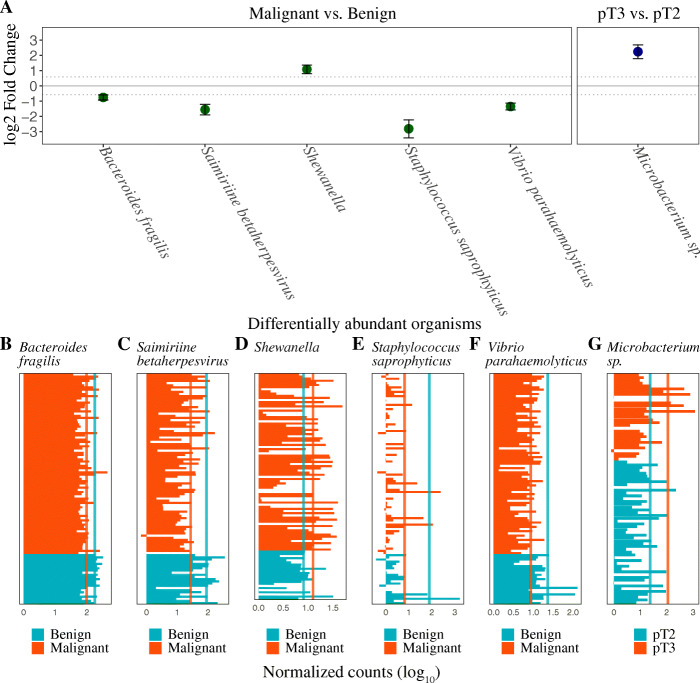
**A** Differentially abundant microorganisms (adjusted *p* value < 0.01) identified in the comparisons between malignant and benign tissue samples, and pathological T-stage 3 vs. 2 (pT3 vs. pT2). Error bars correspond to the standard error of the log_2_ fold change. Malignant tissue sample was associated with over-abundance of *Shewanella*, and under-representation of *Bacteroides fragilis*, *Saimiriine betaherpesvirus*, *Staphylococcus saprophyticus*, and *Vibrio parahaemolyticus*. Pathologically advanced T3 stage tumors were associated with significantly increased abundances of *Microbacterium* species. Dotted line corresponds to a log_2_ fold change cut-off value of |0.58|. **B**–**H** Normalized microbial read counts (*x*-axis) for each patient sample (*y*-axis) of *B. fragilis* (**B**), *S. betaherpesvirus* (**C**), *Shewanella* (**D**), *S. saprophyticus* (**E**), *V. parahaemolyticus* (**F**), and *Microbacterium sp*. (**G**). Values are given on a log_10_ scale. Vertical lines denote mean values. Missing bars represent a normalized count value of zero

We next performed differential abundance analysis between benign (*n* = 23) and malignant tissue (*n* = 83) samples in order to see whether any particular organism or group of organisms were under- or over-represented in PCa tissue in the discovery cohort. We used a cut-off criteria of an adjusted *p* value less than 0.01, a log_2_ fold change greater than |0.58|, and a normalized mean count greater than 10 across all samples, for assigning differentially abundant species. Using these criteria, we found the genus *Shewanella* to be significantly over-abundant in the malignant as compared to benign prostate tissue samples. Similarly, we found four microbial organisms (including a virus) to have significantly lower abundances in the malignant tissue as compared to the benign tissue (Fig. 2, Table 3). These included *Bacteriodes fragilis*, *Staphylococcus saprophyticus*, *Vibrio parahaemolyticus*, and *Saimiriine betaherpesvirus*.

**Table 3:** Differentially abundant microbial reads in various comparisons in the discovery cohort

Organism name	Base mean	Log_2_ fold change	lfcSE	Wald statistic	***p*** value	***p*** adj
**Malignant vs. benign tissue (discovery cohort)**						
*Shewanella*	11.61	1.08	0.28	3.83	0.0001	0.0042
*Bacteroides fragilis*	115.60	−0.76	0.17	−4.33	1.45e−05	0.0008
*Saimiriine betaherpesvirus*	40.72	−1.56	0.34	−4.56	4.99e−06	0.0004
*Staphylococcus saprophyticus*	21.99	−2.82	0.59	−4.77	1.80e−06	0.0002
*Vibrio parahaemolyticus*	11.78	−1.36	0.22	−6.08	1.15e−09	5.19e−07
**Pathologic T-stage 3 vs. 2 (discovery cohort)**						
*Microbacterium sp.*	56.12	2.24	0.45	4.92	8.35e−07	0.0049

Note: Showing all differentially abundant microorganisms with an adjusted *p* value less than 0.01 and a base mean greater than 10. Base mean, mean of the normalized count across all samples. *lfcSE* standard error of the log_2_ fold change, *p-adj* Benjamini-Hochberg adjusted *p* value

**Fig. 3:**
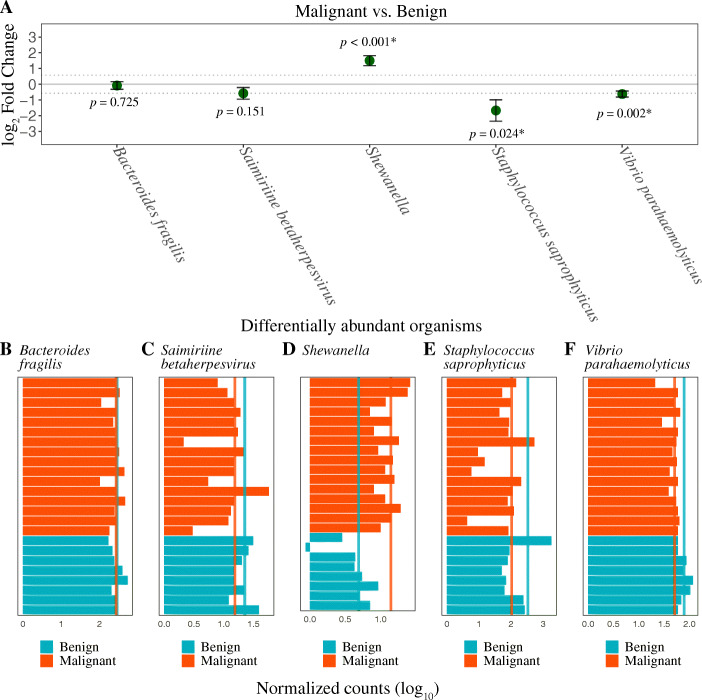
**A** Differentially abundant organisms between malignant and benign tissue samples identified in the discovery cohort were tested for their differential abundance in an independent validation cohort. Of the five organisms tested, three showed a significant difference in their abundance (adjusted *p* value < 0.05; marked by asterisk). Malignant tissue sample was associated with significant over-abundance of *Shewanella*, and significant under-representation of *Staphylococcus saprophyticus*, and *Vibrio parahaemolyticus*, as compared to the benign tissue samples. *Bacteroides fragilis* and *Saimiriine betaherpesvirus* showed a trend similar to that seen in the discovery cohort, although the differences did not reach statistical significance in the validation cohort. Error bars correspond to the standard error of the log_2_ fold change. Dotted line corresponds to a log_2_ fold change cut-off value of |0.58|. **B**–**H** Normalized microbial read counts (*x*-axis) for each patient sample (*y*-axis) of *B. fragilis* (**B**), *S. betaherpesvirus* (**C**), *Shewanella* (**D**), *S. saprophyticus* (**E**), and *V. parahaemolyticus* (**F**). Values are given on a log_10_ scale. Vertical lines denote mean values

In order to validate the association between PCa and the five microbial species identified as differentially abundant between benign and malignant tissue samples in the discovery cohort, we tested for their differential abundance between benign (*n* = 8) and malignant tissue (*n* = 16) samples using an independent validation cohort (Additional file 1: Fig. S1). Of the five species identified, we were able to validate the differential abundance of three species (*Shewanella*, *V. parahaemolyticus*, and *S. saprophyticus*) at a significance cut-off adjusted *p* value less than 0.05 and a fold change greater than 1.5. *Shewanella* was significantly over-abundant, whereas *V. parahaemolyticus* and *S. saprophyticus* had significantly lower abundance in the malignant samples as compared to the benign samples (Fig. 3, Table 4), corroborating the results seen in the discovery cohort. Differences in *B. fragilis* and *S. betaherpesvirus* counts between benign and malignant tissue samples were not statistically significant in the smaller validation cohort but did show a trend towards lower abundance in the malignant samples, similar to that seen in the discovery cohort (Fig. 3, Table 4). The observed presence of differentially abundant (both under- and over-represented) microbial species in malignant tissue suggests to a possible microbial dysbiosis associated with PCa.

**Table 4:** Validation of species identified as differentially abundant between malignant and benign tissue samples in the discovery cohort

Organism name	Base mean	Log_2_ fold change	lfcSE	Wald statistic	***p*** value	***p*** adj
**Malignant vs. benign tissue (validation cohort)**						
*Shewanella*	10.97	1.49	0.32	4.64	3.33e−06	1.6e−05*
*Bacteroides fragilis*	276.86	−0.08	0.24	−0.35	0.7253	0.7253
*Saimiriine betaherpesvirus*	17.75	−0.58	0.37	−1.54	0.1214	0.1517
*Staphylococcus saprophyticus*	180.95	−1.67	0.68	−2.44	0.0145	0.0242*
*Vibrio parahaemolyticus*	60.13	−0.62	0.19	−3.30	0.0009	0.0024*

Note: Species identified as differentially abundant in the discovery cohort were tested for their differential abundance in the validation cohort. Significance was determined at an adjusted *p* value less than 0.05 (marked by asterisk). Base mean, mean of the normalized count across all samples. *lfcSE* standard error of the log_2_ fold change, *p-adj* Benjamini-Hochberg adjusted *p* value

**Fig. 4:**
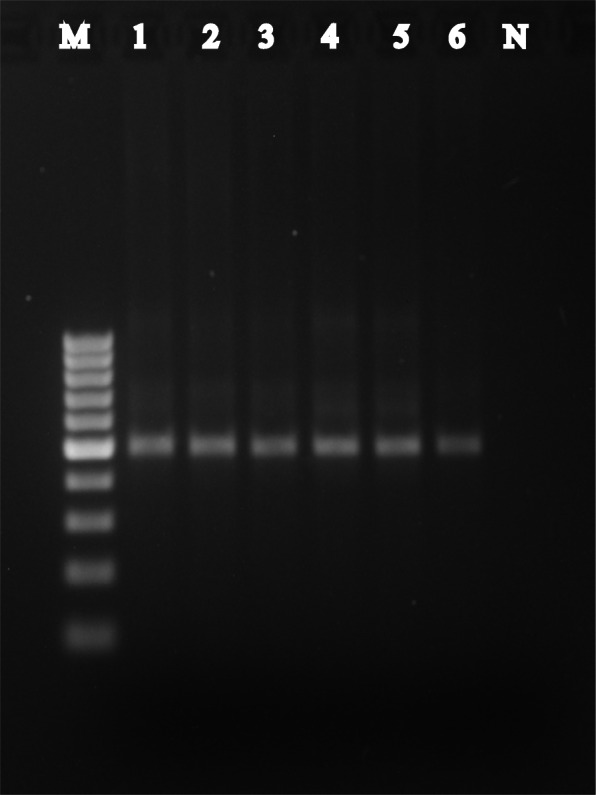
Reverse transcription PCR detection of *Bacteroides fragilis* showing expected band around 495bp. Lanes 1, 2, and 3 are benign tissue samples. Lanes 4, 5, and 6 are malignant samples. M 100bp marker. N negative control

To demonstrate the validity of the microbial read processing pipeline, we performed reverse transcription PCR using species specific primers to detect *B. fragilis* in the RNA samples. We detected the presence of *B. fragilis* in all the prostate tissue samples tested (*n* = 6, Fig. 4), which was also validated by Sanger sequencing of PCR amplicons (Additional file 1: Table S2), thereby confirming the presence of the species in the tissue as opposed to errors in bioinformatics sequence annotation. Together with the detection of all five species (*Shewanella*, *V. parahaemolyticus*, *S. saprophyticus*, *B. fragilis*, and *S. betaherpesvirus*) in the validation cohort, these findings support that our results are most likely to be “true” signals from the prostate tissue microbiome.

**Fig. 5:**
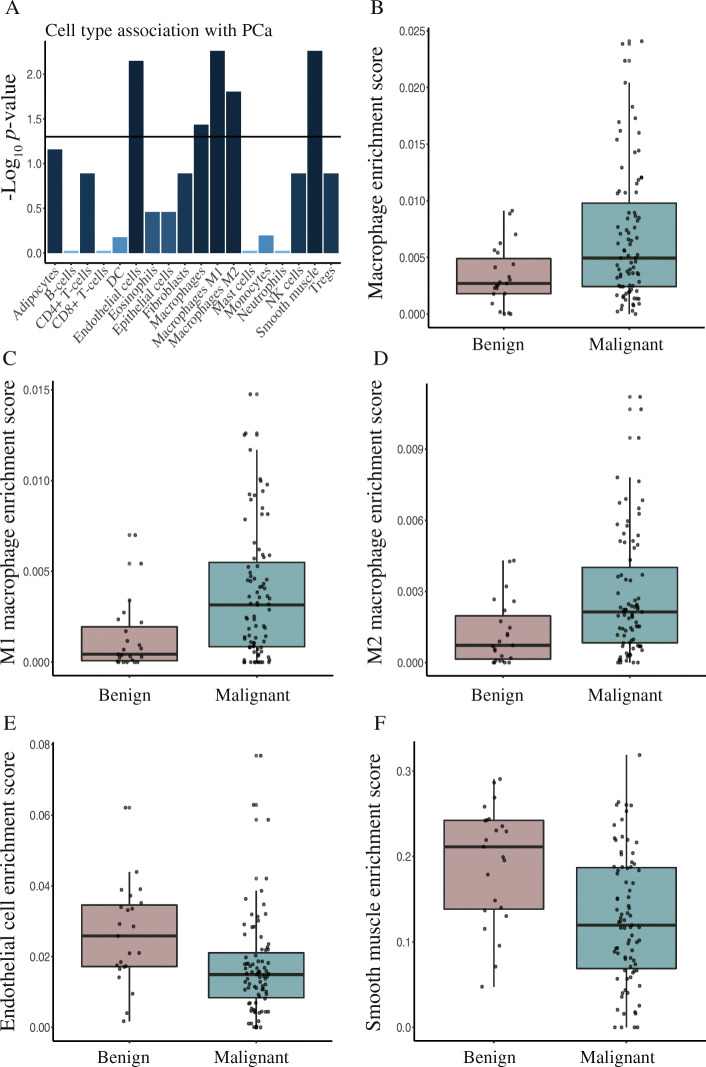
**A** Bar chart showing association between cell types and PCa. A Wilcoxon test was used to determine significant associations. Horizontal line corresponds to a *p* value cut-off of 0.05. FDR corrected *p* values are reported. Macrophages, M1 macrophages, M2 macrophages, endothelial cells, and smooth muscle showed a significant association with PCa. **B**–**F** Boxplots showing differences in the enrichment scores between benign vs. malignant tissue samples for **B** macrophages, **C** M1 macrophages, **D** M2 macrophages, **E** endothelial cells, and **F** smooth muscle

Furthermore, to test whether malignant prostate tissue samples were associated with an altered immune cell composition that might be linked with microbial dysbiosis, we performed cell type enrichment analysis in the discovery cohort comparing malignant (*n*=83) vs. benign (*n*=23) tissue samples. We observed significant (FDR < 0.05) associations between PCa and the presence of macrophages (including both M1 and M2 macrophages), endothelial cells, and smooth muscle cells (Fig. 5A). Macrophages had significantly higher enrichment scores in the malignant (*n* =83) as compared to the benign (*n* = 23) tissue samples (Fig. 5B–D), suggesting a possible link between macrophage mediated immune regulation and microbial dysbiosis in PCa.

### Associations between microbial presence and PCa aggressiveness

Next, we looked for overall differences in species diversity between less vs. more aggressive PCa using alpha diversity estimates and further used differential abundance analysis to characterize microbial species that were more likely to be associated with aggressive PCa. For these analyses, we made subsets of the malignant tissue samples in the discovery cohort based on several clinicopathological factors (Table 1) including Gleason scores, pre-op PSA levels, pathologic T-stage, and BCR status. Due to the limited sample size, these analyses were not performed in the validation cohort.

For 83 malignant tissue samples, we compared samples having a low (≤7, *n* = 63) Gleason score with those having a high (>7, *n* = 20) Gleason score. We observed an overall increase in the species diversity in the high as compared to the low Gleason score tissue samples (Additional file 1: Fig. S2A). However, there were no differentially abundant organisms present in either low or high Gleason score groups at an adjusted *p* value less than 0.01, likely reflecting our stringent filtering criteria for the DGE analyses which filtered species with very low counts. Similarly, we did not find any significantly under- or over-represented species when we compared malignant tissue samples from patients having a low (≤10 ng/ml, *n* = 39) vs. high (>10 ng/ml, *n* = 39) pre-op PSA levels, while some alpha diversity estimates suggested higher species richness in tumors from patients with higher pre-op PSA levels (Additional file 1: Fig. S2B). For the comparison between pathological T2 (*n* = 51) vs. T3 (*n* = 31) stages, we observed an overall increased species diversity in pT3 vs. pT2 (Additional file 1: Fig. S2C). Differential abundance analysis between these groups identified *Microbacterium sp.* to have significantly increased abundances in the more advanced pT3 stage tissue samples (adjusted *p* < 0.01, Table 3) compared to the less advanced pT2 tissue (Fig. 2). This suggests an association between *Microbacterium sp*. and PCa disease progression; however, further investigations are warranted.

In order to test whether microbial dysbiosis could be associated with BCR, we compared malignant tissue samples (*n* = 81) from patients in the discovery cohort who had BCR (*n* = 28) to those who did not (BCR-free; *n* = 53). However, in this cohort, we did not find any organisms to be significantly differentially abundant in the patients who had BCR compared to those who did not, but some alpha diversity estimates suggested higher species richness in tumors from patients suffering BCR (Additional file 1: Fig. S2D).

### Host transcriptional regulation in PCa with high vs. low counts of *Shewanella*, *Vibrio parahaemolyticus*, or *Staphylococcus saprophyticus*

**Fig. 6:**
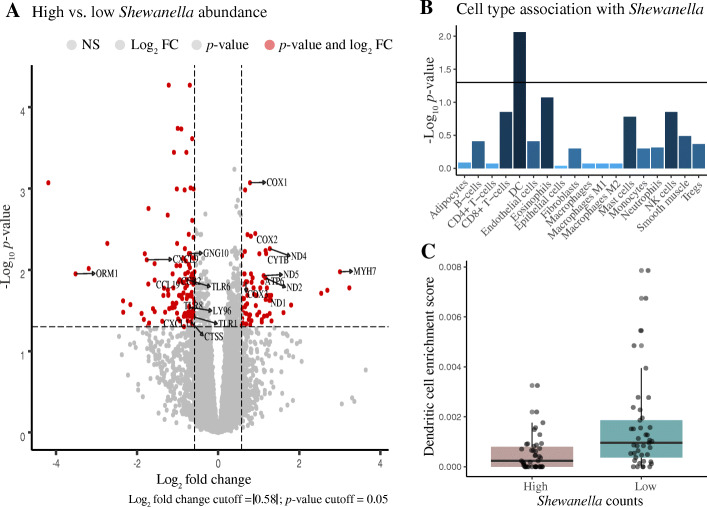
**A** Volcano plot showing differentially expressed host genes in the comparison between high vs. low *Shewanella* counts group. Genes involved in significant KEGG pathways (Table 5) are also labelled in the plot. Benjamini-Hochberg corrected *p* values are reported. NS not significant. FC fold change. **B** Bar chart showing association between cell types and *Shewanella*. A Wilcoxon test was used to determine significant associations. Horizontal line corresponds to a *p* value cut-off of 0.05. FDR corrected *p* values are reported. Dendritic cells (DC) showed a significant association with *Shewanella*. **C** Boxplot showing differences in the enrichment score for DC between high vs. low *Shewanella* counts group

To test whether malignant tissue samples harbouring high (*n* = 42) vs. low (*n* = 41) species counts of *Shewanella*, or low (*n*=41) vs. high (*n*=42) counts of *V. parahaemolyticus* and *S. saprophyticus,* respectively, were associated with altered host gene expression, we performed DGE analyses for the tissue samples in the discovery cohort. For the analyses comparing high vs. low *Shewanella* count groups, we identified 501 genes to be differentially expressed between the two groups at an adjusted *p* value < 0.05. Of these, 71 genes were upregulated, whereas 131 genes were downregulated in the high as compared to the low *Shewanella* count group at a fold change cut-off of |1.5| (Fig. 6A, Additional file 2: Table S3). KEGG pathway analyses of the genes upregulated in the high vs. low *Shewanella* count groups showed significant (FDR < 0.05) enrichment of oxidative phosphorylation and metabolic pathways (Table 5), indicating increased energy utilisation by the malignant tissue samples with high abundance of *Shewanella*. Furthermore, genes that were downregulated were significantly enriched for Toll-like receptor signaling pathway (Table 5), indicating a downregulated immune system in prostate tumors with high abundance of *Shewanella*.

**Table 5:** Results from Kyoto Encyclopedia of Genes and Genomes (KEGG) pathway analysis. Upregulated and downregulated host genes identified from the comparison between high vs. low *Shewanella* count group were used for KEGG pathway analysis

**Significant KEGG pathways in upregulated genes**			
**Pathway**	**Fold enrichment**	***p*** **value**	***p*** **adj**
Oxidative phosphorylation	21.88	1.32e−11	4.74e−10
Parkinson’s disease	20.49	2.56e−11	4.74e−10
Metabolic pathways	3.25	1.77e−05	2.19e−04
Cardiac muscle contraction	17.63	1.38e−04	0.0012
Alzheimer’s disease	7.87	0.0029	0.0215
Huntington’s disease	6.89	0.0046	0.0289
**Significant KEGG pathways in downregulated genes**			
**Pathway**	**Fold enrichment**	***p*** **value**	***p*** **adj**
Toll-like receptor signaling pathway	12.61	1.31e−05	8.91e−04

*p-adj p* value corrected for false discovery rate

While no significant KEGG pathways were observed for the analyses of *S. saprophyticus*, pathways relating to olfactory transduction, retinol metabolism, steroid hormone biosynthesis, and cytochrome P450 mediated drug and xenobiotic metabolism pathways were significantly enriched in the malignant samples having low (vs. high) abundance of *V. parahaemolyticus* (Additional file 3: Table S4 and Additional file 1: Table S5). The enrichment of, e.g., drug metabolism and steroid hormone biosynthesis pathways, could suggest that patients with low *V. parahaemolyticus* counts might be more prone to developing drug resistance. Furthermore, genes involved in pathways relating to focal adhesion, mineral absorption, regulation of actin cytoskeleton, ECM-receptor interaction, vascular smooth muscle contraction, and cardiomyopathy were significantly downregulated in the malignant samples having low (vs. high) *V. parahaemolyticus* counts (Additional file 3: Table S4 and Additional file 1: Table S5). This may indicate altered regulatory interactions between the TME and PCa cells in tumors with low abundance of *V. parahaemolyticus*.

### Host cell type enrichment in PCa with high vs. low counts of *Shewanella*, *Vibrio parahaemolyticus*, or *Staphylococcus saprophyticus*

Finally, to test whether malignant tissue samples harbouring high vs. low species counts of *Shewanella*, and low vs. high counts of *V. parahaemolyticus* and *S. saprophyticus*, respectively, were associated with an altered immune/stromal architecture, we performed cell type enrichment analysis using xCell [37]. For the comparison between high (*n* = 42) vs. low (*n* = 41) *Shewanella* counts group within the malignant samples, we observed a significant (FDR < 0.05) inverse association between *Shewanella* genera counts and dendritic cells (DC, Fig. 6B) with the high *Shewanella* count group having a lower DC enrichment score compared to the low count group (Fig. 6C). This corroborates the downregulation of immune related genes observed in the DGE analyses and indicates an important role for DCs in PCa pathophysiology.

Further, malignant samples having a low (*n* = 41) vs. high (*n* = 42) *V. parahaemolyticus* counts had a significantly (FDR < 0.05) lower enrichment score for adipocytes, fibroblasts, and smooth muscle (Additional file 1: Fig. S3A-C) and a significantly higher enrichment score for monocytes, B cells, CD8+ T cells, mast cells, M2 macrophages, T regulatory cells (Additional file 1: Fig. S3D-I), and neutrophils (borderline significant, not shown), suggesting altered host immunity and dysregulated TME architecture, as also indicated by the downregulation of genes and pathways involved in, e.g., focal adhesion, regulation of actin cytoskeleton, and ECM-receptor interaction, that were identified in our DGE and KEGG pathway analyses, respectively. However, for *S. saprophyticus*, we did not observe any significant associations with the host stroma or immune cell types.

## Discussion

Over the past decade, a more holistic approach towards understanding human diseases has led us to the holobiont nature of many diseases, including cancer. The role of the human microbiome in cancer has increasingly garnered attention from the scientific community, both in terms of its direct and indirect roles in carcinogenesis, as well as its diagnostic and prognostic potential [42–44]. So far, no single pathogenic species has been causally associated with PCa, but a growing body of evidence suggests that changes to the healthy microbiota (i.e., microbial dysbiosis) could play a major role in disease occurrence, progression, and/or treatment outcome (reviewed by 14). Thus, it is important to map the microbiota of the benign and diseased prostate tissue environment to better understand the link between PCa and the prostate microbiome.

A number of factors can determine the resident human microbial ecosystem, including diet, ethnicity, and geography which often distinguishes populations [45, 46]. We here aimed to investigate the role of prostate microbiota in PCa development or progression in a cohort of Danish men and provide a comprehensive analysis of the benign (adjacent normal) and diseased (malignant) prostate tissue microbiome. Using a metatranscriptomic approach, we found several bacterial species to have very high relative abundances in the prostate tissue among Danish men who had PCa (discovery cohort). These included genera such as *Enterobacter*, *Acinetobacter*, *Streptococcus*, and *Escherichia* which have also been reported to be abundant in prostate tissue in other studies [12, 47], but are also known to be common contaminants in sequencing-based microbiome studies [15, 41]. Thus, whether these are true representative species of the prostate microbiome or whether these are introduced during procedural handling requires further investigation.

Additionally, we identified *Mycobacterium sp.* and *Salmonella enterica* at high relative proportions in this Danish PCa discovery cohort. Of the species that have not previously been reported as contaminants in sequencing studies, we found *S. enterica*, *C. jejuni*, and *C. difficile* to have relatively high abundances in the prostate tissue of PCa patients, perhaps more accurately reflecting the “true” microbial ecology of the prostate.

While many studies have reported *Propionibacterium acnes* (renamed *Cutibacterium acnes*) to be abundant in prostate tissue [12, 47], *P. acnes* did not represent a major taxa across all samples in this patient cohort. Thus, our results do not indicate an association between PCa and the pro-inflammatory effects of *P. acnes* as suggested in other studies [11, 48, 49]. It is noteworthy that *Propionibacterium* is a common contaminant in sequencing studies [15, 40, 41], and most sequencing-based microbiome studies have failed to account for this possibility, perhaps explaining the contradictory results that we observed. In addition, factors such as differences in patient cohorts, sample types, sample handling, sample size, and the data analysis methodology employed can all influence which microbial species are detected, partly explaining discrepancies in the results between our study and previous studies.

### Microbial dysbiosis between malignant and benign tissue samples identified through differential abundance analysis

We observed an overall reduction in the species diversity within the malignant as compared to the benign tissue samples from patients with PCa in both the discovery cohort from Denmark and the validation cohort from France. Corroborating these findings, our differential abundance analysis comparing malignant vs. benign tissue samples revealed four species to have significantly lower abundances in the malignant tumor tissue. Validity of these findings and of the microbial annotation pipeline was demonstrated by performing a reverse transcription PCR which successfully detected the presence of *Bacteroides fragilis* in all tested samples, as also confirmed by Sanger sequencing of the PCR amplicons. The presence of these species were also validated in an independent cohort in which two of the four species also showed significantly lower abundance in the malignant vs. benign tissue samples.

Interestingly, the observed lower abundances of *B. fragilis* in the malignant as compared to the benign tissue samples in the discovery cohort is in contrast to the suspected role played by this organism in colon and rectal cancers [50]. For example, rectal cancer was associated with higher abundances of *B. fragilis* in the tumor but not non-tumor tissue samples [51]. Such a result is however not surprising, given an organism can potentially have different roles in different organs. For example, increased tissue abundances of the genus *Lactobacillus* is reported to be associated with breast cancer [52], whereas a lower abundance of lactobacilli in the cervico-vaginal microbiome is thought to be associated with ovarian cancer [53].

The only microbe showing significantly higher abundance in malignant tissue as compared to the benign tissue in the discovery cohort and validated in the independent cohort belonged to the genus *Shewanella.* This genus has not been reported in PCa previously, although higher abundances of *Shewanella* have been reported in the colon mucosa of patients with rectal and distal cancers as opposed to proximal colorectal cancer [54]. This could suggest to an association between *Shewanella* and at least some cancer types, making it a highly interesting candidate for further studies.

### Altered host immune regulation associated with increased *Shewanella* abundance

Differential expression and KEGG pathway analyses between tissue samples in the discovery cohort having high vs. low *Shewanella* count revealed significant downregulation of key immune related genes and pathways in the high *Shewanella* count group, including Toll-like receptors (TLR) such as TLR1, TLR6, and TLR8. Human TLRs are expressed by leukocytes such as dendritic cells (DC) and forms part of the innate immune system that is involved in pathogen recognition [55]. Downregulation of the TLR signaling pathway in the malignant tissue samples with high *Shewanella* count could indicate pathogen associated immune dysregulation. However, no tissue inflammation within the prostate was evident in the histology for a majority of the patients with malignant tumor (77/83, data not shown). Thus, it may be unlikely that *Shewanella* induces tissue inflammatory responses that could lead to PCa development. It is more likely that decreased immune activity in the malignant tumor of these patients might provide a conducive tumor microenvironment for the growth of this bacteria.

Decreased enrichment scores for dendritic cells in the high vs. low *Shewanella* group also suggest the existence of a downregulated adaptive immune-system, since DCs have a crucial role in antigen presentation to T cells [56]. Decreased DC activity within these malignant tissue samples could perhaps enable immune evasion by *Shewanella*. However, we did not find any significant differences in other immune cell types between high vs. low *Shewanella* count group, which is also consistent with the absence of histologically visible tissue inflammation in most patients. While the observed link between *Shewanella* and DC remains to be investigated in more depth, our results suggest that presence of high levels of *Shewanella* in the tumor may increase vulnerability towards cancer immunotherapy that targets TLR in DCs [57]. Further characterization of the TLRs within the malignant tissue is necessary to validate whether these TLRs are in fact associated with DC.

### Altered host gene expression associated with decreased *V. parahaemolyticus* abundance

Differential gene expression and KEGG pathway analyses between tissue samples in the discovery cohort having low vs. high *V. parahaemolyticus* count revealed significant upregulation of genes involved in olfactory transduction pathways in the low *V. parahaemolyticus* count group. Genes encoding twenty-two different olfactory receptors (OR) were upregulated in this group. ORs are G-protein-coupled receptors that detect odorants (chemosensation). They are mainly expressed in the olfactory epithelium, but have also been shown to be over-expressed in prostate tumor cells [58], where they could play a role in promoting cancer invasiveness and metastasis [59]. Interestingly, five of the OR genes that we identified (OR51T1, OR51S1, OR51G2, OR51A7, and OR51F2) were also significantly upregulated in abiraterone/enzalutamide resistant VCap xenograft tumors as compared to tumors prior to castration; Pre-Cx [60]. Of note, OR51T1 and OR51S1 were also significantly upregulated in the castration resistant PCa (CRPC) vs. Pre-Cx VCap tumors in the same study. Taken together with the upregulation of genes involved in steroid hormone biosynthesis and drug metabolism pathways in low vs. high *V. parahaemolyticus* counts group as observed in our study, these findings suggest that patients with a low abundance of *V. parahaemolyticus* might be more likely to progress towards a castration resistant phenotype and/or might be more prone to developing drug resistance following treatment with abiraterone/enzalutamide.

### Species associated with advanced disease stage

Comparisons of malignant tissue microbiome from patients with pathological T-stages pT2 and pT3 revealed significantly higher abundances of *Microbacterium sp.* in pT3 prostate cancer samples in the discovery cohort. *Microbacterium sp.* are nosocomial infectious agents that have also been reported in the blood of, e.g., lung, and pancreatic cancer patients, although disease stage of the underlying malignancy was not significantly correlated with *Microbacterium sp.* infection rate [61]. While we detected *Microbacterium sp.* within malignant tissue samples in the validation cohort (mean number of reads = 54.3, standard deviation = 29.1), we did not test for its differential abundance due to the limited sample size in this cohort. Thus, further validation of its association with PCa using a larger cohort is warranted. Whether these could be a potential novel target for therapeutic intervention in patients having more advanced pathological tumor stages also remains to be explored.

Our study has some limitations. First, we did not test for possible sources of contamination during procedural handling as all analyses were based on existing total RNAseq data. However, we took a more conservative approach and excluded samples that had very high abundances of taxa that are known to be contaminating sequences from reagent kits based on previously published studies. Additionally, by using metatranscriptomic analysis, it is possible that we missed microbial taxa that are not actively transcribing or have a low level of transcription, which could have been captured by metagenomic analysis. Our study did not yield microbial read counts in sufficient numbers to enable elucidation of the functional relevance of the prostate microbiome and its association with PCa. Finally, the lack of non-PCa control samples limits our conclusions to differences between benign (adjacent normal) and malignant tumor tissue. However, collection of truly normal prostate tissue is ethically challenging and also difficult for men in this age group (mean approx. 65 years), where occult PCa and/or benign prostatic hyperplasia is commonly observed [62].

## Conclusions

In conclusion, we show that prostate cancer is associated with dysbiosis of the prostate microbial ecosystem with reduced overall species diversity in the malignant as compared to the benign tissue samples. Differential abundances (both higher and lower) of certain species in the malignant prostate tissue could provide diagnostic and/or prognostic potential in the future. In particular, *Shewanella* genera might be associated with malignant transformation of the prostate tissue that could be facilitated by a decreased host immune response. Down-regulation of genes involved in TLR signaling pathway and decreased enrichment of DCs were both associated with increased *Shewanella* counts and could be candidate targets for future immunotherapy. Upregulation of genes involved in olfactory transduction, drug metabolism, and steroid hormone biosynthesis pathways was associated with decreased *V. parahaemolyticus* counts and might be indicative of patients who are more susceptible to developing treatment resistance. *Microbacterium sp.* is an interesting candidate for further investigations on its association with PCa aggressiveness. Our results thus support the existence of an important biological link between the prostate microbiota and PCa development/progression. These aspects should be further investigated in future research including histologically visualizing the localization of these bacteria as well as relevant host cells (e.g., epithelial/tumor, stromal, and immune cells) within intact prostate tissue specimens using in situ detection methodologies.

## Supplementary Information

**Additional file 1.** Supplementary Tables and Figures. This file contains Tables S1, S2, S5 and Figures S1-S3.

**Additional file 2: Table S3.** Differentially expressed host genes identified in the malignant tissue samples having high vs. low *Shewanella* counts.

**Additional file 3: Table S4.** Differentially expressed host genes identified in the malignant tissue samples having low vs. high *Vibrio parahaemolyticus* and *Staphylococcus saprophyticus* counts, respectively.

**Additional file 4: Table S6.** Normalised microbial count data for the discovery cohort.

**Additional file 5: Table S7.** Normalised microbial count data for the validation cohort.

**Additional file 6: Table S8.** Normalised host gene count data for the discovery cohort - benign samples.

**Additional file 7: Table S9.** Normalised host gene count data for the discovery cohort - malignant samples 1-42.

**Additional file 8: Table S10.** Normalised host gene count data for the discovery cohort - malignant samples 43-83.

## Data Availability

As the requirement for patient consent was waived in the current study, we do not have permission to deposit the full raw and individual level clinical data in a (controlled access) repository for the discovery cohort. Instead, raw data and individual level clinical data are available from the corresponding author upon reasonable request due to Danish data protection laws. Under Danish law, access to this data will require (1) Ethical approval of the new research project, which has to be applied for to the relevant Danish authority by the data owner (corresponding author of this work, Aarhus University Hospital) on behalf of the PI/collaborator of the new project. (2) After ethical approval has been obtained, a data sharing agreement for the new project is required between the data owner (corresponding author, Aarhus University Hospital) and the PI/collaborator of the project and their institutions. The processed dataset used and/or analyzed in the discovery cohort is included within the manuscript as Additional file 4: Table S6 and Additional files 6, 7 and 8: Tables S8-S10, respectively. The dataset corresponding to the validation cohort is available online at Gene Expression Omnibus under the accession GSE115414 (https://www.ncbi.nlm.nih.gov/geo/query/acc.cgi?acc=GSE115414) [17]. Processed microbial read counts for the validation cohort are available as Additional file 5: Table S7.

## Abbreviations

BCR: Biochemical recurrence
CRPC: Castration resistant prostate cancer
DC: Dendritic cells
DGE: Differential gene expression
FDR: False discovery rate
KEGG: Kyoto Encyclopedia of Genes and Genomes
OR: Olfactory receptor
PCa: Prostate cancer
Pre-op PSA: Pre-operative prostate specific antigen
RNAseq: RNA sequencing
RP: Radical prostatectomy
TLR: Toll-like receptor
TME: Tumor microenvironment
