# Supplementary material for: Microbiota of the prostate tumor environment investigated by whole-transcriptome profiling
Source: Genome Med. 2022 Jan 25;14:9. doi: 10.1186/s13073-022-01011-3 (PMC8787950; doi:10.1186/s13073-022-01011-3)
Supplement: Supplementary file 1 — Additional file 1. Supplementary Tables and Figures. This file contains Tables S1, S2, S5 and Figures S1-S3. [file 13073_2022_1011_MOESM1_ESM.docx]

Additional file 1 of

Microbiota of the prostate tumor environment investigated by whole-transcriptome profiling

Paul Vinu Salachan^1,2^, Martin Rasmussen^1,2^, Jacob Fredsøe^1,2^, Benedicte Ulhøi^3^, Michael Borre^2,4^, Karina Dalsgaard Sørensen^1,2^

^1^Department of Molecular Medicine, Aarhus University Hospital, 8200 Aarhus N, Denmark

^2^Department of Clinical Medicine, Aarhus University, 8200 Aarhus N, Denmark

^3^Department of Pathology, Aarhus University Hospital, 8200 Aarhus N, Denmark

^4^Department of Urology, Aarhus University Hospital, 8200 Aarhus N, Denmark

Tables

Table S1. Wilcoxon test results comparing alpha diversity estimates for various comparisons.

| **Comparisons** | **Observed** | | **Chao1** | | **ACE** | | **Shannon** | | **Simpson** | | **Inverse Simpson** | |
| --- | --- | --- | --- | --- | --- | --- | --- | --- | --- | --- | --- | --- |
|  | ***W*** | ***P*** | ***W*** | ***P*** | ***W*** | ***P*** | ***W*** | ***P*** | ***W*** | ***P*** | ***W*** | ***P*** |
| **Discovery cohort** | | | | | | | | | | | | |
| Malignant vs. Benign | 1909 | 1.5e-12 | 1909 | 1.5e-12 | 1909 | 1.5e-12 | 1730 | 1.7e-08 | 1539 | 4.5e-05 | 1539 | 4.5e-05 |
| High vs. Low Gleason | 0 | 1.2e-10 | 0 | 1.2e-10 | 0 | 1.2e-10 | 46 | 3.1e-09 | 194 | 2.1e-05 | 194 | 2.1e-05 |
| High vs. Low PSA | 148 | 5.7e-09 | 84 | 9.1e-14 | 44 | 1.9e-16 | 650 | 1.6e+00 | 703 | 3.4e+00 | 703 | 3.4e+00 |
| pT3 vs. pT2 | 0 | 2.5e-13 | 0 | 2.5e-13 | 0 | 2.5e-13 | 287 | 9.0e-06 | 453 | 7.6e-03 | 453 | 7.6e-03 |
| BCR vs. BCR-free | 16 | 3.4e-12 | 156 | 3.6e-08 | 85 | 4.2e-10 | 545 | 3.0e-01 | 601 | 9.7e-01 | 601 | 9.7e-01 |
| **Validation cohort** | | | | | | | | | | | | |
| Malignant vs. Benign | 128 | 1.6e-05 | 128 | 1.6e-05 | 128 | 1.6e-05 | 42 | 1.1e+00 | 28 | 1.6e-01 | 28 | 1.6e-01 |

*W*, Wilcoxon test statistic. *P*, *p* value corrected for family wise error rate using the Bonferroni method.

Table S2. Validation of PCR amplicons by Sanger sequencing.

| **SampleID** | **Scientific Name** | **Max Score** | **Total Score** | **Query Cover** | **E Value** | **Percent Identity** | **Accession Length** | **Accession** |
| --- | --- | --- | --- | --- | --- | --- | --- | --- |
| M2 | *Bacteroides fragilis* | 706 | 706 | 98% | 0 | 91.15 | 1436 | NR_119164.1 |
| M5* | *Bacteroides fragilis* | 577 | 577 | 90% | 2.00E-164 | 88.83 | 1436 | NR_119164.1 |
| M11 | *Bacteroides fragilis* | 521 | 521 | 85% | 1.00E-147 | 89.33 | 1436 | NR_119164.1 |
| B10 | *Bacteroides fragilis* | 544 | 544 | 98% | 2.00E-154 | 90.35 | 1436 | NR_119164.1 |
| B13 | *Bacteroides fragilis* | 601 | 601 | 93% | 1.00E-171 | 88.94 | 1436 | NR_119164.1 |
| B22 | *Bacteroides fragilis* | 521 | 521 | 82% | 1.00E-147 | 89.57 | 1436 | NR_119164.1 |

Shown are the identity of *B. fragilis* to assembled contigs, except in one case (asterisk) where identity to the reverse read is shown.

Table S5. Results from Kyoto Encyclopedia of Genes and Genomes (KEGG) pathway analysis. Up- and down-regulated host genes identified from the comparison between low vs. high Vibrio parahaemolyticus count group were used for KEGG pathway analysis.

| **Significant KEGG pathways in up-regulated genes** | | | |
| --- | --- | --- | --- |
| **Pathway** | **Fold enrichment** | ***p* value** | ***p*-adj** |
| Olfactory transduction | 5.577841663 | 7.01E-11 | 6.94E-09 |
| Drug metabolism - cytochrome P450 | 8.926038062 | 4.89E-04 | 0.020410426 |
| Metabolism of xenobiotics by cytochrome P450 | 8.202305246 | 7.22E-04 | 0.020410426 |
| Drug metabolism - other enzymes | 10.99584399 | 9.86E-04 | 0.020410426 |
| Chemical carcinogenesis | 7.587132353 | 0.00103083 | 0.020410426 |
| Ascorbate and aldarate metabolism | 14.9869281 | 0.002185536 | 0.033229337 |
| Steroid hormone biosynthesis | 8.720841785 | 0.002349549 | 0.033229337 |
| Retinol metabolism | 7.903262868 | 0.003368396 | 0.041683901 |
| Pentose and glucuronate interconversions | 12.26203209 | 0.003910476 | 0.04301524 |
|  | | | |
| **Significant KEGG pathways in down-regulated genes** | | | |
| **Pathway** | **Fold enrichment** | ***p* value** | ***p*-adj** |
| Dilated cardiomyopathy | 4.939569161 | 2.79E-08 | 5.88E-06 |
| cGMP-PKG signaling pathway | 3.455394816 | 1.60E-07 | 1.69E-05 |
| Vascular smooth muscle contraction | 3.91965812 | 2.53E-07 | 1.78E-05 |
| Calcium signaling pathway | 3.172013833 | 4.60E-07 | 2.43E-05 |
| Hypertrophic cardiomyopathy (HCM) | 4.47960928 | 1.78E-06 | 7.53E-05 |
| Focal adhesion | 2.756264448 | 6.35E-06 | 2.23E-04 |
| cAMP signaling pathway | 2.647041847 | 3.08E-05 | 9.27E-04 |
| Arrhythmogenic right ventricular cardiomyopathy (ARVC) | 4.23724236 | 4.14E-05 | 0.00109091 |
| Adrenergic signaling in cardiomyocytes | 3.006694272 | 4.98E-05 | 0.001167746 |
| Mineral absorption | 4.963203463 | 1.32E-04 | 0.002787404 |
| Protein digestion and absorption | 3.474242424 | 1.58E-04 | 0.003036343 |
| Neuroactive ligand-receptor interaction | 2.128623002 | 3.44E-04 | 0.006050787 |
| Proteoglycans in cancer | 2.293 | 7.54E-04 | 0.012245937 |
| Regulation of actin cytoskeleton | 2.183809524 | 0.00138483 | 0.020871363 |
| ECM-receptor interaction | 3.012151067 | 0.001886739 | 0.026525358 |
| Cardiac muscle contraction | 3.202920635 | 0.002011402 | 0.026525358 |

p-adj, *p* value corrected for false discovery rate.

Figures


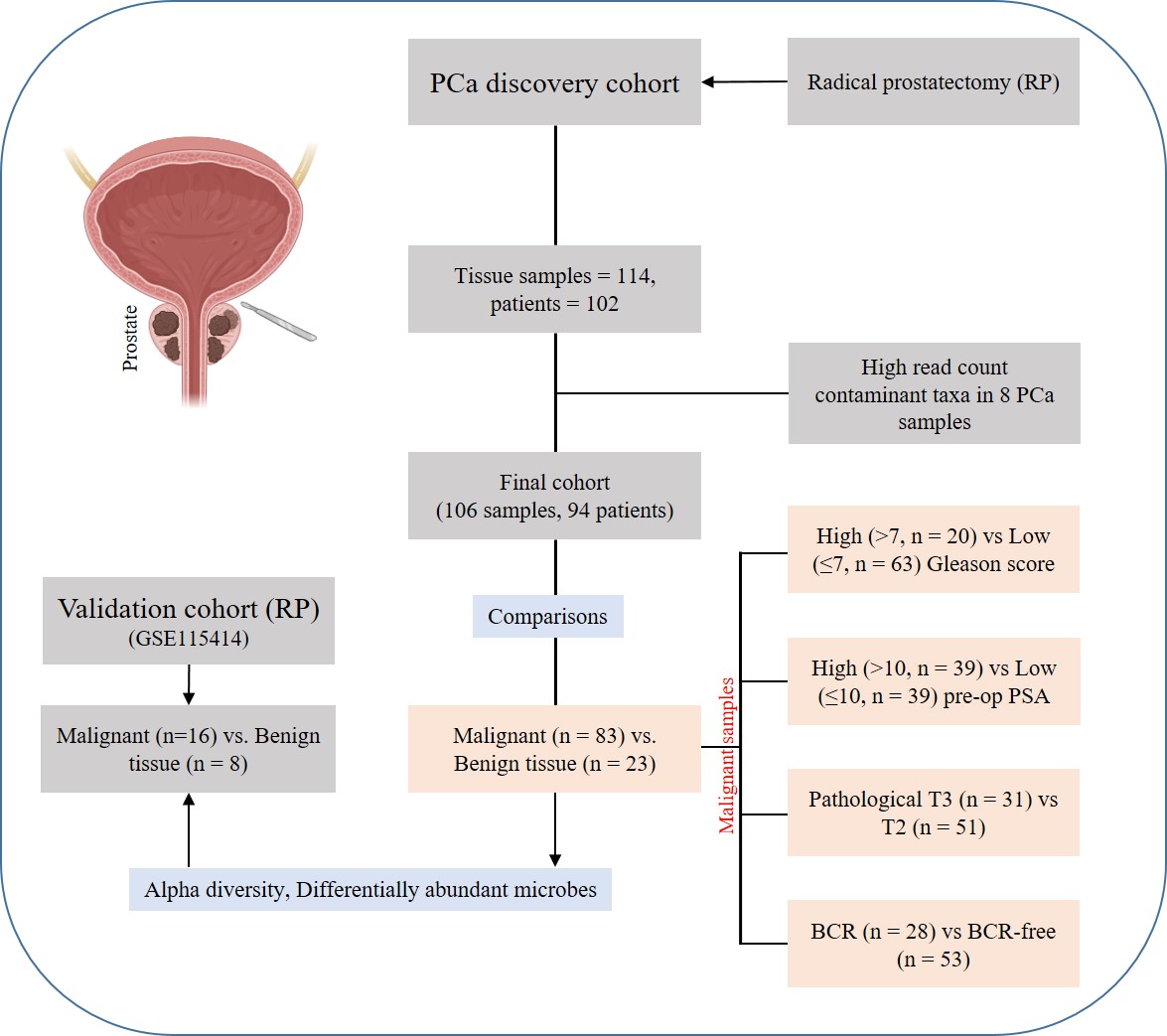


Figure S1. Overview of the patient cohort and the samples used in various comparisons. Exclusion of samples were performed due to the presence of very high read counts with very high contributions from taxa that are known to be tissue/reagent contaminants from previous studies. PCa, prostate cancer. Pre-op PSA, pre-operative prostate specific antigen. BCR, biochemical recurrence. Image partly made using Biorender.com


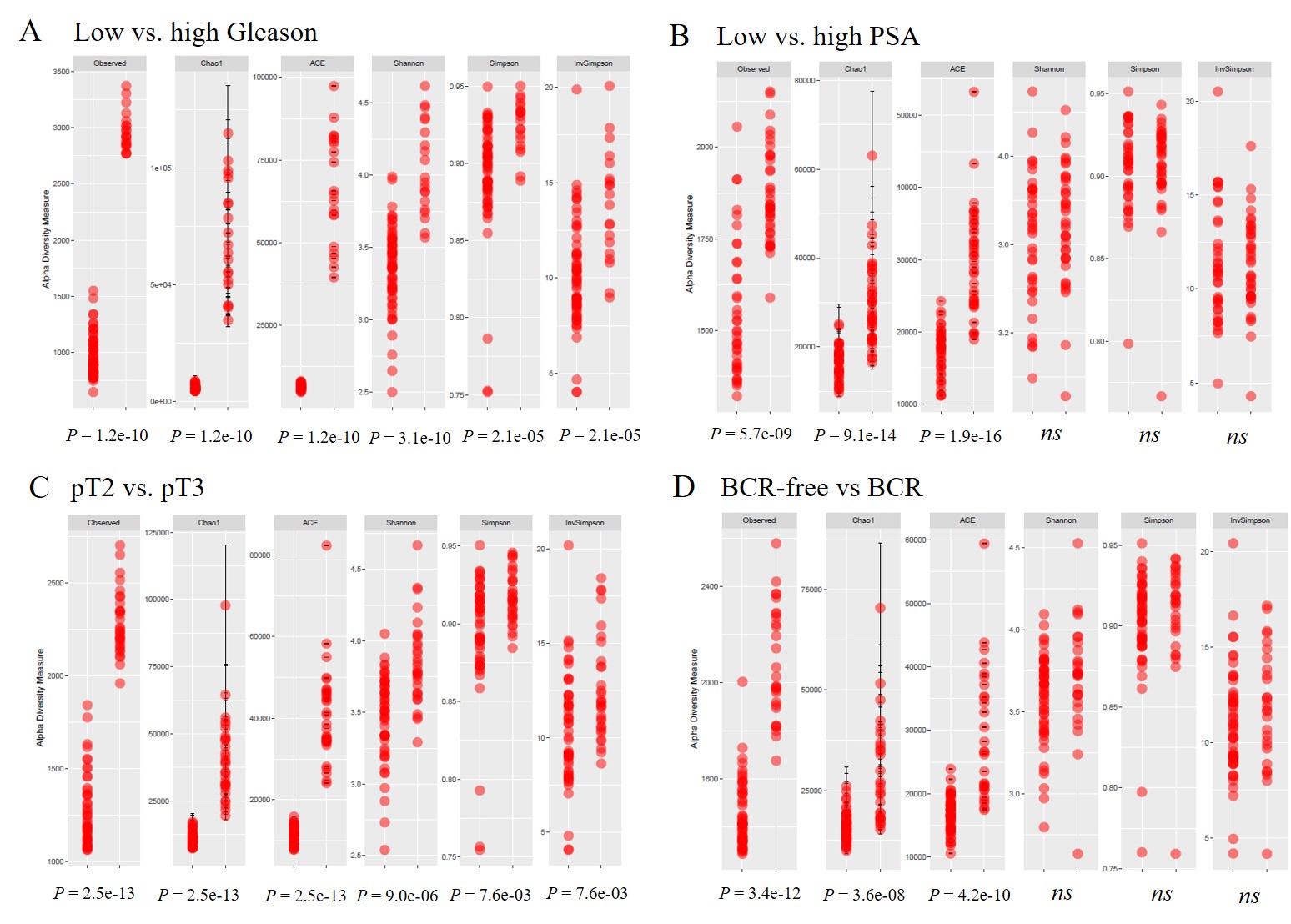


Figure S2. Differences in alpha diversity between less vs. more aggressive PCa based on A) pathological T-stage (pT2 vs.pT3), B) Gleason score (low vs. high), C) prostate specific antigen (low vs. high PSA) and D) biochemical recurrence (BCR-free vs. BCR). For each of the diversity measure, diversity within the less aggressive samples are shown first. A Wilcoxon rank sum test was used to compare the diversity estimates between the two groups and statistical significance was considered at a p value cut-off of 0.05. The more aggressive tissue samples had higher species diversity compared to the less aggressive tissue samples, although significance varied depending on the metric used. ns, not significant.


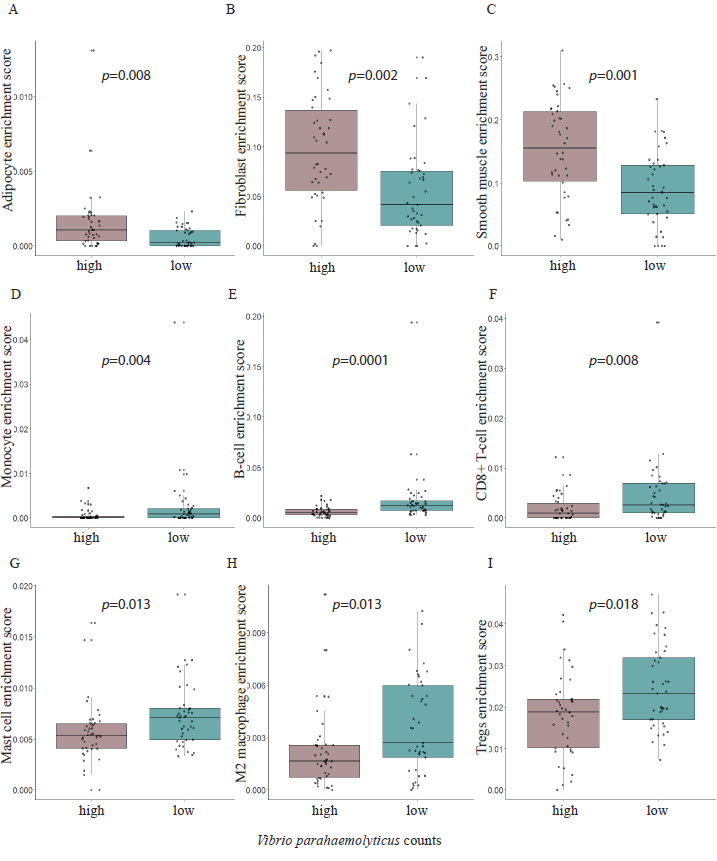


Figure S3. Boxplots showing differences in the enrichment scores between high vs. low Vibrio parahaemolyticus counts group for A) Adipocytes, B) Fibroblasts, C) Smooth muscle, D) Monocytes, E) B-cells, F) CD8+ T-cells, G) Mast cells, H) M2 macrophages, and I) T regulatory cells.
